# Evolution of the Young’s Modulus of Al-7Si-4Cu Alloy with Increasing Temperature by Various Strengthening Approaches

**DOI:** 10.3390/ma19091831

**Published:** 2026-04-29

**Authors:** Hongyu Wang, Jingyi Hu, Tong Gao, Hongfu Su, Shushuai Liu, Xiangfa Liu

**Affiliations:** 1 Key Laboratory for Liquid-Solid Structural Evolution and Processing of Materials, Ministry of Education, Shandong University, 17923 Jingshi Road, Jinan 250061, China; 202434213@mail.sdu.edu.cn (H.W.); 202320616@mail.sdu.edu.cn (J.H.); 15634051221@163.com (H.S.); xfliu@sdu.edu.cn (X.L.); 2 State Key Laboratory of Coatings for Advanced Equipment, Shandong University, 17923 Jingshi Road, Jinan 250061, China

**Keywords:** Al-Si-Cu alloy, Young’s modulus, high temperature, strengthening

## Abstract

Despite the crucial role of Young’s modulus in the structural performance of Al alloys, the effects of common strengthening approaches on its evolution, particularly at elevated temperatures, remain largely unexplored. In this study, an Al-7Si-4Cu alloy was modified by hot deformation, micro-alloying with 0.3 wt.% Sc, alloying with 4 wt.% Ni, and reinforcement with 0.8 vol.% Al_2_O_3_ nanoparticles. The effects of these strengthening approaches on the microstructure and the evolution of Young’s modulus from room temperature to 350 °C were examined. It was found that the Young’s modulus of the alloys decreased with the increase in temperature, while this tendency is much more obvious when the temperature exceeds 250 °C. The results showed that hot deformation markedly refines the α-Al grains while the Young’s modulus stays largely unchanged. The Sc addition leads to the formation of the W phase but has no significant effect on the Young’s modulus. In contrast, the addition of Ni substantially increases the Young’s modulus through the formation of Al_3_CuNi intermetallic particles, with the Young’s modulus increasing from 72.15 to 76.47 GPa. With the addition of Al_2_O_3_ particles, the decreasing magnitude of Young’s modulus is optimized when the temperature is higher than 250 °C. This work may be referred to when designing high-modulus Al alloys by considering the utilization of various strengthening concepts.

## 1. Introduction

Al-Si alloys are widely used in the industrial fields due to their excellent castability, wear resistance, and heat resistance, while various alloying elements are often employed to improve their mechanical properties [1,2]. For instance, the addition of Cu can significantly enhance the strength of Al-Si alloys, which is attributed to the precipitation of the θ′-Al_2_Cu nanometric particles during the heat treatment [3,4,5]. Consequently, Al-Si-Cu alloys such as A390 and ZL107 are used in lightweight applications like the automotive industry, exhibiting significant weight advantages over Ti and Fe alloys [6,7,8,9]. However, a key drawback of Al-based alloys is their low Young’s modulus. The Young’s modulus of pure Al is typically only about 72 GPa, which is roughly one-third that of Fe [10].

Over the years, common strengthening mechanisms for improving the performance of Al alloys included grain refinement, second-phase, and dispersion strengthening. Researchers have utilized approaches such as hot deformation, alloying, and particle reinforcement to effectively enhance alloy strength. Hot deformation is a convenient approach that promotes dynamic recrystallization through plastic deformation at elevated temperatures, thereby achieving grain refinement and improvements in strength and ductility [11,12,13,14]. In contrast to hot deformation, alloying enhances properties by introducing strengthening elements [15,16]. Among various alloying elements, Sc is a particularly effective micro-alloying element for Al alloys, and it facilitates the formation of nanoscale coherent L1_2_-Al_3_Sc precipitates within the Al matrix during heat treatment procedures [17]. When a certain content of Cu and Sc co-exists in an Al alloy, Al_3_Sc may act as heterogeneous nucleation sites for θ′-Al_2_Cu, which in turn enhances the thermal stability of θ′-Al_2_Cu [18,19]. With respect to the high-temperature performance, studies have shown that adding Ni to Al-Si-Cu alloys promotes the formation of multiple heat-resistant intermetallic compounds, thereby enhancing their high-temperature strength [20,21,22]. In parallel, particle reinforcement is another effective approach that enhances mechanical properties by incorporating ceramic particles into the alloy matrix. Various ceramic particles, such as Al_3_BC, AlN, Al_2_O_3_ and TiB_2_, have been shown to provide a synergistic improvement in both strength and Young’s modulus [23,24]. Among them, Al_2_O_3_ is widely employed due to its excellent cost–performance ratio and good Young’s modulus [25]. However, the influences of the abovementioned approaches on the Young’s modulus at different temperatures can rarely be studied.

Based on the above considerations, in this study we systematically investigated the effects of multiple strengthening approaches on the microstructure and Young’s modulus of a typical cast Al-7Si-4Cu alloy. The Al-7Si-4Cu alloy is close to the nominal content of a ZL107 Al alloy, which is widely used in the fields of aerospace, automotive manufacturing, and precision instruments, etc. The strengthening approaches in this study included hot extrusion, Sc micro-alloying, Ni alloying, and Al_2_O_3_ particle reinforcement, which are commonly applied for strengthening Al-Si and Al-Si-Cu alloys [26,27,28,29]. The evolution of Young’s modulus with increasing temperature from room temperature to 350 °C was examined in detail. The results provide valuable reference for understanding the temperature-dependent evolution behavior of the Young’s modulus of Al alloys.

## 2. Materials and Methods

### 2.1. Alloy Preparation Approaches

Al-7Si-4Cu was designed as the base alloy, while Al-7Si-4Cu-0.3Sc (in wt.% unless otherwise stated), Al-7Si-4Cu-4Ni and Al-7Si-4Cu-0.8 vol.% Al_2_O_3_ alloys were prepared to test the influence of micro-alloying, alloying, and particle reinforcing on the Young’s modulus of the alloy. All of these alloys were prepared by using commercial pure Al (99.8%), commercial pure Si (99.9%), Al-50Cu, Al-2Sc and Al-30Ni master alloys, provided by Shandong Mai Ao Jing Advanced Materials Co., Ltd., Linyi, China. Among them, the Al_2_O_3_ particles required during the alloy preparation process originate from the Al-Al_2_O_3_ material [30]. The initial materials were heated in a clean clay-bonded graphite crucible by a resistance furnace when they were completely melted. C_2_Cl_6_ was used for slag removal and melt degassing. The melt was finally poured into a preheated cast-iron mold. In addition, a portion of the Al-7Si-4Cu alloy melt was poured into a graphite mold and hot-extruded at 450 °C with an extrusion ratio of 16:1. All of the alloys then underwent a T6 treatment: solution treatment at 520 °C for 8 h, water quenching, aging at 170 °C for 8 h, and air cooling. The alloys correspond to different strengthening approaches, and their designations are listed in Table 1.

### 2.2. Microstructural Characterization and Performance Tests

X-ray diffraction (XRD, Rigaku D/max-rB, Tokyo, Japan) was performed for phase identification and texture analysis using Cu Kα radiation (λ = 1.5406 Å) at 40 kV and 100 mA, with a 2θ scanning range from 10° to 90°. For scanning electron microscopy (SEM), the sample surfaces were etched with a 0.4 vol% HF aqueous solution and then examined using a field-emission SEM (FE-SEM, Hitachi SU-70, Tokyo, Japan) operated at 15 kV. The instrument was equipped with an energy-dispersive X-ray spectrometer (EDS, HORIBA, EX-250, Tokyo, Japan) for chemical microanalysis. Transmission electron microscopy (TEM) was conducted on an FEI Talos F200X microscope (Hillsboro, OR, USA) to acquire bright-field (BF), high-angle annular dark-field (HAADF), and high-resolution TEM (HRTEM) images. Electron backscatter diffraction (EBSD) analysis was performed using a JEOL JSM-7800F SEM (Japan Electron Optics Laboratory Co., Ltd., Tokyo, Japan) to characterize the grain size, crystallographic orientation, grain boundary (GB) distribution, and recrystallized fraction (RF).

The samples were machined to the dimension of 120 × 15 × 10 mm^3^ to test their Young’s modulus using an IET-1000P dynamic Young’s modulus tester (Zhuosheng, Luoyang, China). For high-temperature tests, a heating rate of approximately 3 °C/min was applied, and the samples were held at the target temperature for 10 min prior to measurement. The reported modulus value at each temperature is the average of five measurements.

## 3. Results

### 3.1. Microstructural Analysis

#### 3.1.1. AC Alloy

**Figure 1:**
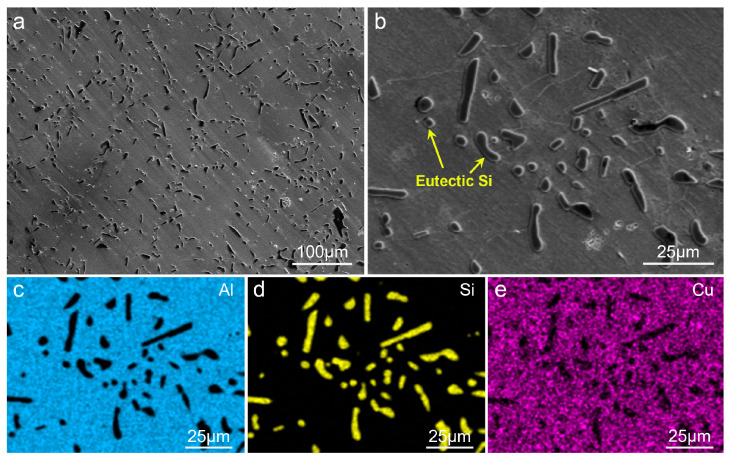
Microstructures of the T6-treated AC alloy: (**a**,**b**) SEM images; (**c**–**e**) EDS mappings.

**Figure 2:**
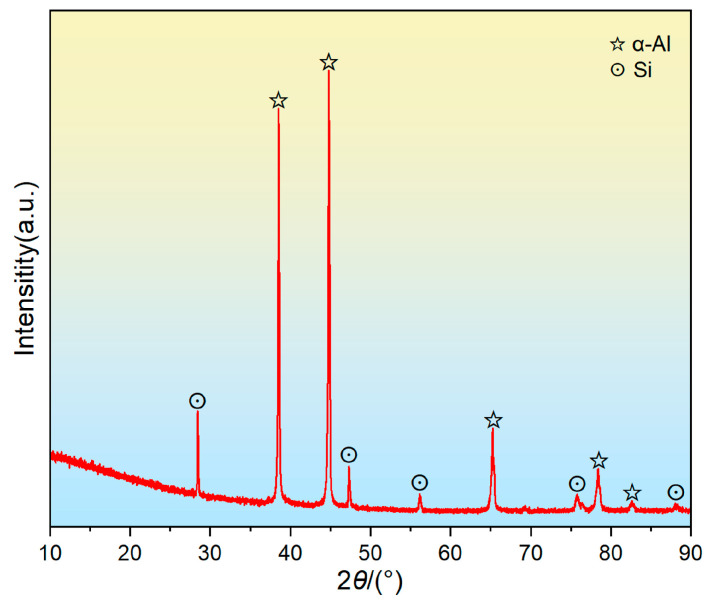
XRD pattern of T6-treated AC alloy.

The microstructures of the T6-treated AC alloy are shown in Figure 1. As can be seen, eutectic Si particles are homogeneously dispersed in the Al matrix (Figure 1a). These eutectic Si particles predominantly exhibit short rod-like and spherical morphologies, as displayed in Figure 1b, which are attributed to the fracture and sphering effect during heat treatment. EDS mappings (Figure 1c–e) clearly reveal the morphology, size, and distribution of the eutectic Si particles. Notably, no obvious Al_2_Cu particles were found in the T6-treated sample, indicating that they have almost totally dissolved into the matrix. Figure 2 shows the XRD pattern of the sample, and the absence of Al_2_Cu diffraction peaks confirms the SEM observation.

**Figure 3:**
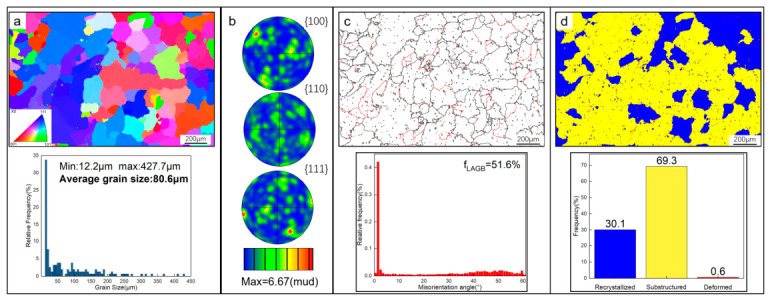
EBSD analysis of the T6-treated AC alloy: (**a**) grain orientation and size distribution of α-Al grains; (**b**) corresponding PFs; (**c**) GB analysis; (**d**) RF analysis.

To investigate the microstructure of the T6-treated AC alloy in depth, EBSD analysis was performed. Figure 3a shows the inverse pole figure (IPF) map of α-Al grains, where different colors represent distinct crystallographic orientations. According to the Euler triangle depicted, the orientations of these α-Al grains are random. The pole figures (PFs) in Figure 3b further confirm the random orientation of the α-Al grains. Additionally, the size distribution of α-Al grains is heterogeneous, with an average size of 80.6 μm (Figure 3a). Figure 3c highlights the grain boundaries of α-Al grains, where low-angle grain boundaries (LAGBs, 2–15°) are denoted in red and high-angle grain boundaries (HAGBs, >15°) are marked in black. LAGBs account for 51.6% of the total grain boundaries, indicating the potential presence of a substantial number of substructural grains in the AC alloy. The RF map of the alloy (Figure 3d) supports this conclusion.

#### 3.1.2. AE Alloy

**Figure 4:**
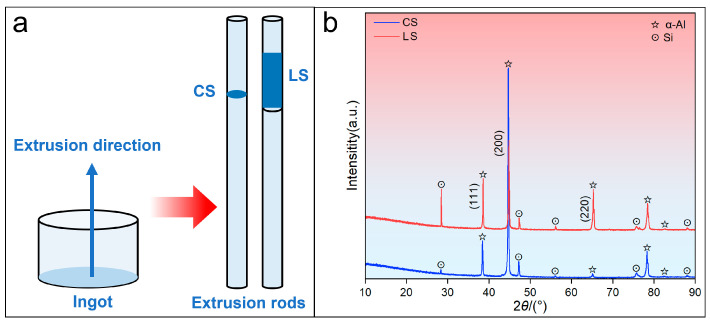
Schematic and XRD patterns of the T6-treated AE alloy: (**a**) Schematic; (**b**) XRD patterns.

Figure 4a presents a schematic of the AE alloy before and after hot extrusion, with the cross-section (CS) and longitudinal section (LS) of the rod indicated. Figure 4b shows the corresponding XRD patterns of the CS and the LS of the T6-treated AE alloy. Compared to the AC alloy, when hot extrusion was applied, the relative intensities of the α-Al diffraction peaks in the AE alloy exhibit significant differences. For example, the (111) and (200) diffraction peaks of α-Al show comparable intensities in the AC alloy, whereas in the AE alloy the (200) peak is notably stronger than the (111) peak. Furthermore, within the AE alloy, the relative intensity of the (220) diffraction peak is markedly higher in the LS than in the CS. These variations in α-Al peak intensities suggest the possible formation of a preferred crystallographic texture of the α-Al grains, resulting from the hot extrusion.

**Figure 5:**
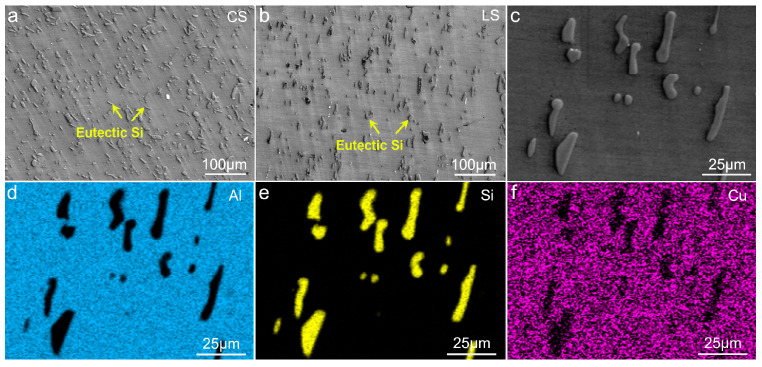
Microstructures of the T6-treated AE alloy: (**a**,**b**) low-magnification SEM images of CS (**a**) and LS (**b**); (**c**) high-magnification SEM image; (**d**–**f**) EDS mappings.

The low-magnified CS and LS microstructures of the T6-treated AE alloy are shown in Figure 5a and Figure 5b, respectively, revealing a uniform distribution of eutectic Si in the matrix. Figure 5c presents the high-magnified LS microstructure, while the corresponding EDS mappings (Figure 5d–f) clearly illustrate the distributions of Al, Si, and Cu, indicating that hot extrusion has not altered the phase composition of the alloy, which is consistent with the XRD results in Figure 4b.

**Figure 6:**
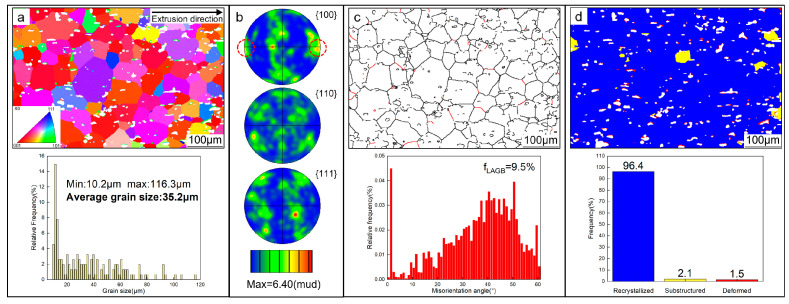
EBSD analysis of the T6-treated AE alloy: (**a**) grain orientation and size distribution of α-Al grains; (**b**) corresponding PFs; (**c**) GB analysis; (**d**) RF analysis.

Figure 6 shows the EBSD analysis results of the T6-treated AE alloy in the LS. As can be seen in Figure 6a, the AE alloy exhibits significantly more refined grains with an average size of 35.2 μm, which is less than half that of the AC alloy. Figure 6b shows the corresponding PFs, in which a slight orientation with <100>//ED forms, as marked by the circles, while no other significant differences can be identified by comparing with the T6-treated AC alloy (Figure 3b). Notably, the proportion of LAGBs drops sharply to only 9.5% (Figure 6c), while the fraction of recrystallized grains rises markedly to 96.4% (Figure 6d), i.e., dynamic recrystallization has obviously occurred during hot deformation.

#### 3.1.3. AS Alloy

**Figure 7:**
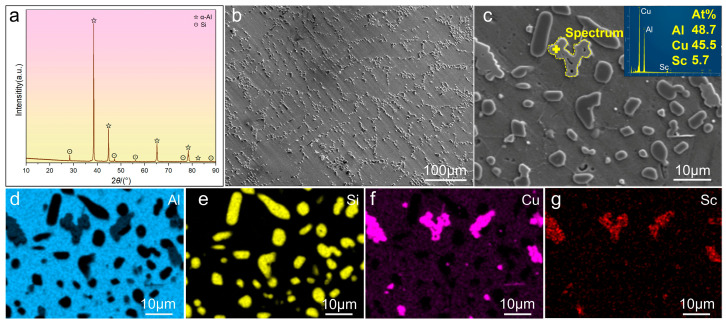
XRD pattern and microstructures of the T6-treated AS alloy: (**a**) XRD pattern; (**b**,**c**) SEM images and spot analysis of AlCuSc phase; (**d**–**g**) EDS mappings.

The XRD pattern and microstructures of the T6-treated AS alloy are shown in Figure 7, i.e., Sc micro-alloying has been applied to the AC alloy. In the XRD pattern (Figure 7a), apart from the diffraction peaks of α-Al and Si, no obvious diffraction peak of the Sc-containing phase was identified. Compared to the AC alloy, the eutectic Si particles are much finer, which can clearly be seen in Figure 7b,c. This may be due to the modifying effect of the Sc element on the eutectic Si, as similar reports can be referred to [31]. In addition, spot analysis (inset of Figure 7c) confirms the presence of a ternary Sc-containing AlCuSc phase, while the EDS mappings (Figure 7d–g) further demonstrate the co-enrichment of Cu and Sc within this AlCuSc phase.

**Figure 8:**
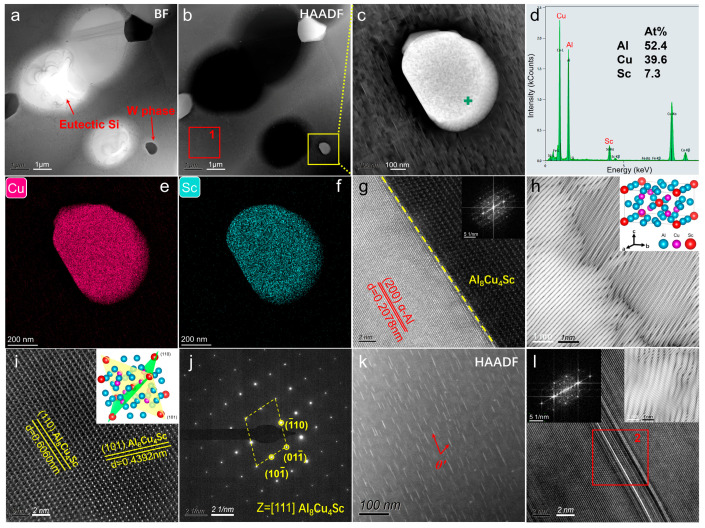
TEM analysis of T6-treated AS alloy: (**a**) BF image; (**b**) HAADF image; (**c**) magnified image of the yellow box in (**b**); (**d**) spot analysis result; (**e**,**f**) EDS mappings of W phase; (**g**) HRTEM image at the interface between α-Al matrix and the W phase; (**h**) IFFT pattern of the interface; (**i**) HRTEM image of the W phase; (**j**) corresponding FFT pattern of (**i**); (**k**) microstructure of θ′-Al_2_Cu nano-precipitates; (**l**) HRTEM image of θ′-Al_2_Cu.

To further analyze and confirm the phase type of the Sc-containing phase, TEM characterization was performed on the AS alloy. Figure 8a,b are the BF and HAADF images of a typical area, and Figure 8c shows an enlarged view of the AlCuSc phase region, clearly revealing its size and morphology. Spot analysis (Figure 8d) yields an approximate atomic ratio of Al:Cu:Sc ≈ 7:5:1, corresponding to the formula Al_7_Cu_5_Sc. This ternary phase is supposed to match the characteristics of the W phase reported in the literature, which has a chemical formula of Al_8−x_Cu_4+x_Sc (0 ≤ x ≤ 2.6) [32,33,34]. EDS mappings of the W phase (Figure 8e,f) further demonstrate the co-enrichment of Cu and Sc, in agreement with the spot analysis. Figure 8g presents an HRTEM image of the interface between α-Al and the W phase, showing a well-defined interface. The measured interplanar spacing of the lattice fringes in α-Al is 0.2078 nm, which corresponds to the (200) plane based on the standard α-Al reference (PDF#00-004-0787). The W phase adopts a ThMn_12_-type structure that crystallizes in the tetragonal system (space group I4/mmm, with lattice parameters of = 0.855–0.862 nm and c = 0.504–0.509 nm [32,35]). The fast Fourier transform (FFT) pattern shown as an insert in Figure 8g is obtained from the interface region. Its inverse fast Fourier transform (IFFT) image is presented in Figure 8h, revealing a non-coherent atomic-scale relationship between α-Al and the W phase. The crystal structure of the W phase is also included in Figure 8h, illustrating its atomic arrangement in the space. Figure 8i shows an HRTEM image of the W phase. The measured interplanar spacings of 0.6060 nm and 0.4392 nm are indexed to the (110) and (101) planes, respectively. Their crystallographic orientations within the unit cell are schematically shown in the insert. Analysis of the corresponding FFT pattern (Figure 8j) confirms that the viewing direction is along the [111] axis zone of the W phase. In addition, a magnified view of region 1 marked by the red box in Figure 8b (Figure 8k) reveals needle-like θ′-Al_2_Cu nano-precipitates dispersed in the matrix. These precipitates, which form during heat treatment, are a common strengthening phase in Al alloys [4,5]. As shown in the HRTEM image of the θ′-Al_2_Cu/α-Al matrix interface (Figure 8l), along with the corresponding FFT and IFFT images from region 2, the precipitates exhibit a highly coherent orientation relationship with the matrix.

#### 3.1.4. AN Alloy

**Figure 9:**
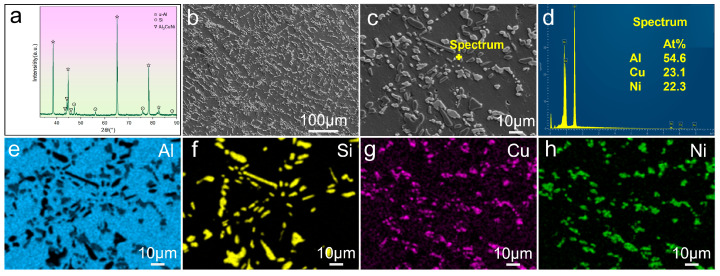
XRD pattern and microstructures of the T6-treated AN alloy: (**a**) XRD pattern; (**b**,**c**) SEM images; (**d**–**h**) EDS results.

Figure 9 illustrates the XRD pattern and microstructures of the T6-treated AN alloy, i.e., Ni alloying has been applied to the AC alloy. As shown in Figure 9a, in addition to α-Al and Si, the diffraction peaks of Al_3_CuNi occur. In addition, the matrix of the AN alloy (Figure 9b) contains not only the gray eutectic Si but also the bright intermetallic particles. EDS spot analysis and mappings were conducted on a typical area (Figure 9c), with the results displayed in Figure 9d–h. Based on the composition of Al, Cu, and Ni, the bright intermetallic is deduced to be Al_3_CuNi, which is consistent with the XRD result. According to the Al-Si-Cu-Ni pseudo-binary phase diagram [36], the solidification pathway of the alloy comprises the following eutectic reactions: L→α-Al+Si, L→α-Al+Al_3_CuNi, and L→α-Al+Al_3_Ni_2_. It is well established that the formation of Ni-containing phases strongly depends on the Cu/Ni ratio in the melt [37,38]. According to our previous work [39], when the ratio between Cu and Ni addition is 1:1, the formation of Al_3_CuNi is reasonable. In addition, with the help of ImageJ software (ImageJ 1.54f), the area fraction of Al_3_CuNi can be obtained. Assuming the morphology and distribution of α-Al, Si, and Al_3_CuNi phases are random, and the corresponding volume fraction of Al_3_CuNi is calculated to be 9.3%.

#### 3.1.5. AO Alloy

**Figure 10:**
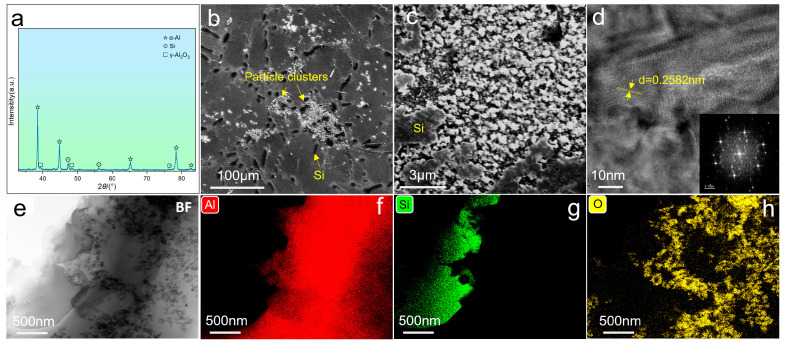
XRD pattern and microstructures of the T6-treated AO alloy: (**a**) XRD pattern; (**b**,**c**) SEM images; (**d**) HRTEM image; (**e**–**h**) EDS mappings.

The XRD pattern and microstructures of the T6-treated AO alloy are shown in Figure 10, i.e., Al_2_O_3_ particle reinforcement has been applied to the AC alloy. The diffraction peaks of γ-Al_2_O_3_ can be identified, as displayed in Figure 10a. It can be found that the Al_2_O_3_ particles prefer to form particle clusters, as shown in Figure 10b,c. Figure 10d is the HRTEM image of a selected Al_2_O_3_ particle, and it is based on the lattice fringe and FFT pattern; the particle was further confirmed to have the γ-type structure. In addition, the image in Figure 10c reveals that some nanometric particles tend to distribute around the eutectic Si particles, which is further confirmed by the TEM image and the corresponding EDS mappings (Figure 10e–h).

### 3.2. Young’s Modulus Test

The Young’s modulus of the abovementioned alloys is measured using the pulse excitation approach via Equation (1) [40]:

(1) $$E\;=\;0.9465\left(\frac{mf^{2}}{b}\right)\left(\frac{L^{3}}{t^{3}}\right)T_{1}$$

where m, f, L, b and t denote the sample mass, fundamental flexural resonant frequency, length, width, and thickness, respectively. The correction factor $T_{1}$, which depends on the sample dimensions and Poisson’s ratio, is given by Equation (2):

(2) $$T_{1}\;=\;1\;+\;6.585\left(1\;+\;0.0752\mu \;+\;0.8109\mu^{2}\right)\left(\frac{t^{2}}{L^{2}}\right)\;-\;0.868\left(\frac{t^{4}}{L^{4}}\right)\;-\;\left[\frac{8.340(1\;+\;0.2023\mu \;+\;2.173\mu^{2})(\frac{t^{4}}{L^{4}})}{1.000\;+\;6.338(1\;+\;0.1408\mu \;+\;1.536\mu^{2})\left(\frac{t^{2}}{L^{2}}\right)}\right]$$

where μ denotes the Poisson’s ratio.

**Figure 11:**
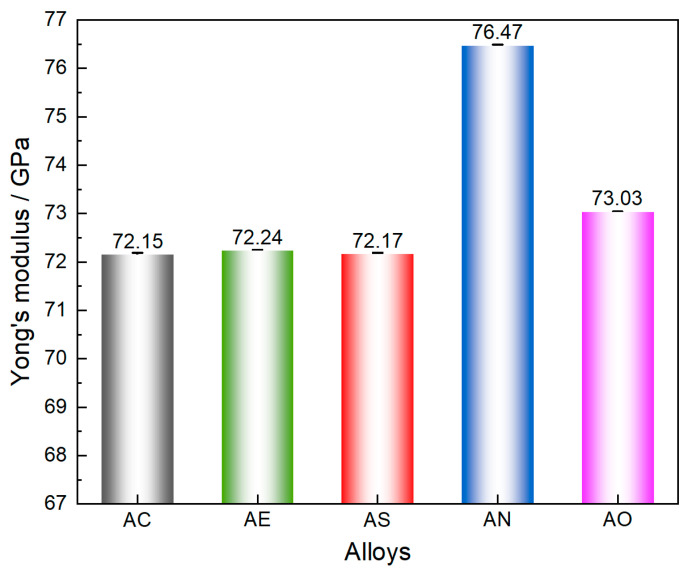
Young’s modulus of the five alloys, tested at room temperature.

The Young’s modulus of the AC, AE, AS, AN, and AO alloys at room temperature (25 °C) is shown in Figure 11. Among them, the modulus of the AC alloy is 72.15 GPa, while the value of the AE alloy (measured on the LS) is 72.24 GPa, exhibiting no obvious difference. As mentioned above, compared to the AC alloy, hot extrusion has significantly altered the grain size of the AE alloy, but its effect on the Young’s modulus is not significant. After Sc micro-alloying, the Young’s modulus of the AS alloy is detected to be 72.17 GPa. Therefore, although Sc micro-alloying has resulted in the formation of the W phase, it also does not observably affect the Young’s modulus. However, with Ni alloying, the Young’s modulus of the AN alloy reaches 76.47 GPa, approximately 6% higher than that of the AC alloy. This increase in Young’s modulus is likely to be attributed to the high-modulus Al_3_CuNi particles, which are present in the fraction of 9.3 vol.% within the AN alloy. In addition, owing to the high modulus of Al_2_O_3_ nanoparticles, the Young’s modulus of the AO alloy is also increased to 73.03 GPa.

**Figure 12:**
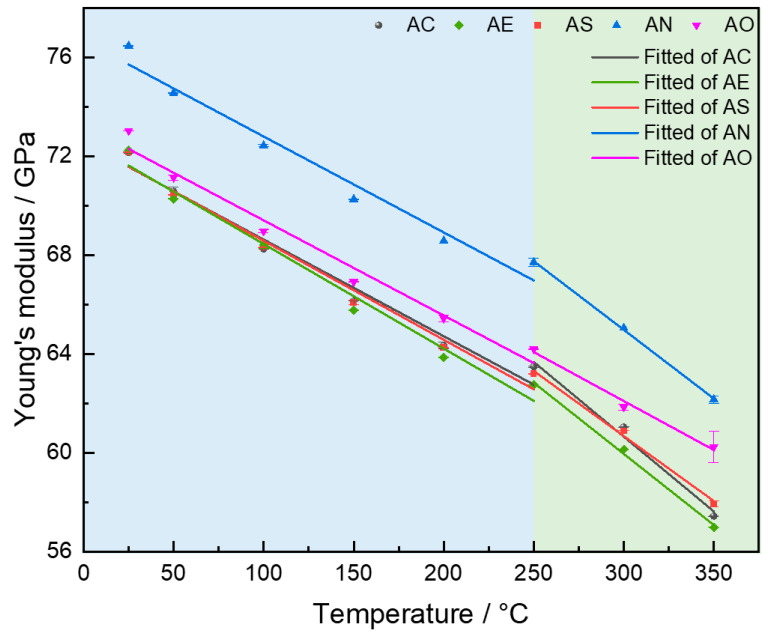
Relationship between the Young’s modulus and temperature for five alloys.

Furthermore, the Young’s modulus of alloys at different elevated temperatures (50, 100, 150, 200, 250, 300, and 350 °C) was characterized, and the values at various temperatures were calculated using Equation (3) [41]:

(3) $$E_{T}\;={\;E}_{0}\frac{f_{T}^{2}}{f_{0}^{2}}(\frac{1}{1\;+\;\alpha \text{∆}T})$$

where $E_{0}$ and $E_{T}$ are the Young’s modulus at room temperature and at temperature T, respectively, and $f_{0}$ and $f_{T}$ are the resonant frequencies at room temperature and at temperature T, respectively. ΔT is the temperature difference between temperature T and room temperature. α is the average linear thermal expansion coefficient, which is regarded as a constant of 23 × 10^−6^ K^−1^ in this study, which is close to the value of pure Al at room temperature. Therefore, the Young’s modulus of five alloys at different temperatures can be calculated, as listed in Table 2. Accordingly, the changing tendency of the Young’s modulus with increasing temperature can be achieved, as shown in Figure 12.

From Figure 12, it is easy to see that the Young’s modulus of all of the samples decreases with the increase in the testing temperature, while the declining rate of AC, AE, AS, and AN alloys seem to accelerate when the temperature exceeds 250 °C. Therefore, the Young’s modulus was fitted using separate lines for the two temperature ranges (before and after 250 °C); the slopes of these fitted lines are listed in Table 3. It can be seen that before 250 °C, the absolute values of the slopes for the five alloys are about 0.04 GPa/°C. However, when the temperature exceeds 250 °C, the absolute value of Young’s modulus’ decreasing rate of these alloys notably increases, while the tendency of the AO alloy shows no difference, i.e., −0.039 GPa/°C for 25–250 °C and −0.040 GPa/°C for 250–350 °C.

## 4. Discussions

As reported in publications [42,43,44,45,46], the Young’s modulus of a material can be predicted using various theoretical models. Among them, the commonly used models include the rule of mixtures (ROM, arithmetic mean), the inverse rule of mixtures (IROM, harmonic mean), and the Halpin-Tsai (H-T) model. The ROM and IROM can provide the upper and lower limits of Young’s modulus and are often used to predict the overall Young’s modulus of multiphase materials. The corresponding expressions [38] are listed as Equations (4) and (5).

(4) $$E_{ROM}=E_{Matrix}V_{Matrix}+E_{Phase1}V_{Phase1}+E_{Phase2}V_{Phase2}+\cdots \;=\sum_{n=0}^{phases}E_{n}V_{n},\;\mathrm{where}\;\sum_{n=0}^{phases}V_{n}=1$$

(5) $$\frac{1}{E_{IROM}}=\frac{V_{Matrix}}{E_{Matrix}}+\frac{V_{Phase1}}{E_{Phase1}}+\frac{V_{Phase2}}{E_{Phase2}}+\cdots =\sum_{n=0}^{phases}\frac{V_{n}}{E_{n}}$$

where E represents the Young’s modulus, V represents the volume fraction, and the subscript ‘0’ denotes the matrix in the equation. Taking the AN alloy, for example, as mentioned above, the volume fraction of Al_3_CuNi is 9.3 vol.%. Therefore, using the Young’s modulus of the AC alloy (72.15 GPa) as the matrix modulus, the upper and lower limits of the Young’s modulus of the AN alloy were calculated to be 82.65 GPa and 76.48 GPa, respectively, according to Equations (4) and (5). Since the Al_3_CuNi phase has simultaneously consumed the Cu element in the AN alloy, therefore the matrix modulus of the AN alloy should theoretically be lower than 72.15 GPa. Consequently, the calculated results are slightly higher than the experimental value (76.47 GPa), which is reasonable to understand.

The H-T model is suitable for predicting the Young’s modulus of metal matrix composites reinforced with discontinuous materials such as particles, whiskers, and 2D (two-dimensional) layered materials. Its expression is as follows: Equations (6) and (7) [46].

(6) $$E_{c}=\frac{E_{m}(1+2S*q*V_{p})}{1-q*V_{p}}$$

(7) $$q=\frac{\left(\frac{E_{p}}{E_{m}}-1\right)}{\left(\frac{E_{p}}{E_{m}}+2S\right)}$$

where $E_{m}$, $E_{p}$, and $E_{c}$ are the Young’s modulus of the matrix alloy, the reinforcement, and the composite, respectively, $V_{p}$ is the volume fraction of the reinforcement, and S is the aspect ratio of the reinforcement. Using this model, the Young’s modulus of the AO alloy in this study can be accurately predicted. By putting the volume fraction of Al_2_O_3_ into the equations, the theoretical Young’s modulus is calculated to be 73.14 GPa. The presence of particle clusters may result in the actual volume fraction of Al_2_O_3_ being lower than the theoretical value, which leads to the predicted value being higher.

As displayed in Figure 12 and Table 3, the Young’s modulus of these alloys does not decrease linearly with increasing temperature but shows a pattern of slower decrease followed by a rapid decline. Taking AC alloy as an example, when the temperature is below 250 °C, the absolute rate of decline is 0.039 GPa/°C. Once the temperature exceeds 250 °C, the absolute rate of decline accelerates significantly, reaching 0.061 GPa/°C. As can be referred to from previous work, when the temperature surpasses 250 °C, the θ′-Al_2_Cu precipitates, rapidly coarsens, and prefers to transform into the θ-Al_2_Cu, leading to the decrease in the particle volume fraction [47,48,49]. Therefore, the rapid decrease in Young’s modulus when the temperature exceeds 250 °C can be associated with this issue. Compared with AC, the Young’s modulus of AE alloy is slightly decreased at elevated temperature, e.g., 63.48 GPa for AC alloy and 62.74 GPa for AE alloy at 250 °C. It may be related to the change in microstructure at elevated temperature, since the AC and AE alloys have different fractions of recrystallized and substructured grains (Figure 3 and Figure 6). A similar tendency can also be found in AS alloy, while the Young’s modulus of AC and AS are still quite similar at each temperature. It may be because the Sc addition is only 0.3%, which obviously does not affect the Young’s modulus. Whilst for the AN alloy, high-modulus Al_3_CuNi particles have formed, which contributes to the enhancing of Young’s modulus at all temperature ranges. Moreover, as can be seen in Figure 12, the AO alloy exhibits a slower decreasing rate in Young’s modulus throughout the entire heating process, especially when the temperature rises from 250 °C to 350 °C. Supposedly, it is attributed to the higher thermal stability of Al_2_O_3_ than the intermetallics in the alloys.

Based on the results of this study and compared with the literature, it can be deduced that although hot deformation or micro-alloying may be an efficient method for alloy strengthening [17,18], their effect on the Young’s modulus is not obvious. Alloying with a proper element to promote the formation of aimed intermetallics, such as the Al_3_CuNi in this study, together with particle reinforcing method are proposed as optional approaches for improving the Young’s modulus. However, the type and content of selected alloying elements and ceramic particles can play an important role. Although this study has focused on the elevated temperature Young’s modulus, there is no doubt that further characterization or calculation is still needed to explain the underlying evolution mechanisms of Young’s modulus with the increase in temperature.

## 5. Conclusions

This study subjected an Al-7Si-4Cu (AC) alloy to hot deformation (AE), 0.3%Sc micro-alloying (AS), 4%Ni alloying (AN), and 0.8 vol.% Al_2_O_3_ reinforcement (AO) to investigate the change in Young’s modulus with the increase in testing temperatures. After T6 treatment, the microstructures and Young’s modulus were characterized. Key findings are summarized as follows:

1. The AC alloy has an average grain size of 80.6 μm, while hot extrusion significantly refines the α-Al grains to 35.2 μm. Following 0.3% Sc micro-alloying, the W (AlCuSc) phase forms in the microstructure, whereas the 4% Ni alloying leads to the formation of Al_3_CuNi. After Al_2_O_3_ addition, particles were found to form clusters.
2. The Young’s modulus of the AC, AE, AS, AN, and AO alloys at room temperature was 72.15, 72.24, 72.17, 76.47, and 73.03 GPa, respectively. Hot extrusion and Sc micro-alloying have negligible effects on the Young’s modulus. The addition of 0.8 vol.% Al_2_O_3_ has slightly increased the Young’s modulus, while the AN alloy exhibits an obvious enhancement.
3. As the temperature increased, the Young’s modulus of the five alloys decreased. The decreasing tendency is much more obvious when the temperature exceeds 250 °C. Ni alloying enables the alloy to maintain a higher Young’s modulus than the base alloy throughout the entire heating stage, while Al_2_O_3_ reinforcement effectively retards the decline in Young’s modulus at elevated temperatures.

## Figures and Tables

**Table 1:** Alloys correspond to different strengthening approaches and their designations.

Alloys	Strengthening Approaches	Designations
Al-7Si-4Cu	-	AC
Al-7Si-4Cu	Hot extrusion	AE
Al-7Si-4Cu-0.3Sc	Sc micro-alloying	AS
Al-7Si-4Cu-4Ni	Ni alloying	AN
Al-7Si-4Cu-0.8 vol.% Al_2_O_3_	Particle reinforcing	AO

**Table 2:** Young’s modulus of five alloys at different temperatures.

Temperature (°C)	Young’s Modulus (GPa)				
	AC	AE	AS	AN	AO
25	72.15 ± 0.02	72.24 ± 0.01	72.17 ± 0.02	76.47 ± 0.02	73.03 ± 0.01
50	70.58 ± 0.17	70.28 ± 0.02	70.44 ± 0.01	74.56 ± 0.01	71.14 ± 0.11
100	68.24 ± 0.01	68.40 ± 0.14	68.32 ± 0.02	72.43 ± 0.05	68.97 ± 0.07
150	66.14 ± 0.01	65.76 ± 0.01	66.08 ± 0.08	70.26 ± 0.02	66.92 ± 0.03
200	64.33 ± 0.13	63.86 ± 0.02	64.25 ± 0.01	68.58 ± 0.00	65.44 ± 0.14
250	63.48 ± 0.02	62.74 ± 0.01	63.19 ± 0.01	67.71 ± 0.16	64.19 ± 0.01
300	61.03 ± 0.01	60.13 ± 0.01	60.90 ± 0.01	65.05 ± 0.00	61.85 ± 0.12
350	57.43 ± 0.02	56.98 ± 0.01	57.93 ± 0.13	62.15 ± 0.15	60.23 ± 0.63

**Table 3:** The slopes of the fitted lines at two temperature ranges from Figure 12.

Alloy	Slopes (GPa/°C)	
	25–250 °C	250–350 °C
AC	−0.039	−0.061
AE	−0.042	−0.058
AS	−0.040	−0.053
AN	−0.039	−0.056
AO	−0.039	−0.040

## Data Availability

The original contributions presented in this study are included in the article. Further inquiries can be directed to the corresponding authors.

## References

[1] Bai Z., Wang Y., Xu X., Ma H., Zhou D., Wang J. (2025). Tensile Fracture Behavior and Friction and Wear Properties of TiB_2_ Particle Reinforced Al-Si Matrix Composites. Ceram. Int..

[2] Niu T., Zhou L., Hu H., Gao W., Sun Y., Zou G., Zu Q., Chen H., Wang P., Peng Q. (2024). Nano-Twinned Silicon in Al-Si Alloys for High Wear-Resistance. Wear.

[3] Bellón B., Haouala S., LLorca J. (2020). An Analysis of the Influence of the Precipitate Type on the Mechanical Behavior of Al-Cu Alloys by Means of Micropillar Compression Tests. Acta Mater..

[4] Shyam A., Roy S., Shin D., Poplawsky J.D., Allard L.F., Yamamoto Y., Morris J.R., Mazumder B., Idrobo J.C., Rodriguez A. (2019). Elevated Temperature Microstructural Stability in Cast AlCuMnZr Alloys through Solute Segregation. Mater. Sci. Eng. A.

[5] Poplawsky J.D., Milligan B.K., Allard L.F., Shin D., Shower P., Chisholm M.F., Shyam A. (2020). The Synergistic Role of Mn and Zr/Ti in Producing θ′/L1_2_ Co-Precipitates in Al-Cu Alloys. Acta Mater..

[6] Wu X., Zhang W. (2024). A Review on Aluminum Matrix Composites’ Characteristics and Applications for Automotive Sector. Heliyon.

[7] Kapranos P., Kirkwood D.H., Atkinson H.V., Rheinlander J.T., Bentzen J.J., Toft P.T., Debel C.P., Laslaz G., Maenner L., Blais S. (2003). Thixoforming of an Automotive Part in A390 Hypereutectic Al–Si Alloy. J. Mater. Process. Technol..

[8] Yu S., Jin Y., Xiong W., Liu Y. (2013). Study on Microstructure and Mechanical Properties of ZL107 Alloy Added with Yttrium. J. Rare Earths.

[9] Li S., Yue X., Li Q., Peng H., Dong B., Liu T., Yang H., Fan J., Shu S., Qiu F. (2023). Development and Applications of Aluminum Alloys for Aerospace Industry. J. Mater. Res. Technol..

[10] Lee Y.-H., Kayani S.H., Lee J.-M., Lee S.-I., Jang J.-I., Cho Y.-H. (2025). Role of Ni in High Elastic Modulus Al-Si-Ni Alloys: Solidification and Microstructure Evolution. Mater. Charact..

[11] Chang K., Miu C., Hung F. (2025). Enhanced Microstructure, Mechanical Properties, and Thermal Stability of Powder Metallurgy Al-Ni-Cu-Fe Alloy through Thermomechanical Processing and Recrystallization. Mater. Today Adv..

[12] Wan S., Su H., Shao B., Zong Y., Shan D., Guo B. (2023). Changes in Microstructure and Mechanical Properties of 2219 Al Alloy during Hot Extrusion and Post-Extrusion Aging. J. Mater. Res. Technol..

[13] Wei Z., Lei Y., Yan H., Xu X., He J. (2019). Microstructure and Mechanical Properties of A356 Alloy with Yttrium Addition Processed by Hot Extrusion. J. Rare Earths.

[14] Miao J., Sutton S., Luo A.A. (2020). Microstructure and Hot Deformation Behavior of a New Aluminum–Lithium–Copper Based AA2070 Alloy. Mater. Sci. Eng. A.

[15] Zhang J., Li Q., Liu G., Zhang X., Wang K., Hu P. (2024). A New Synergy to Overcome the Strength-Ductility Dilemma in Al-Si-Cu Alloy by Adding AlZrNiTi Master Alloy. Mater. Sci. Eng. A.

[16] Fang N., Zou C., Wei Z., Wang H., Zhang X., Chang T. (2021). Effect of Ge and Mg Additions on the Aging Response Behavior and Mechanical Properties of Al-Si-Cu Alloy. Mater. Sci. Eng. A.

[17] Gao Y.H., Cao L.F., Yang C., Zhang J.Y., Liu G., Sun J. (2019). Co-Stabilization of θ′-Al_2_Cu and Al_3_Sc Precipitates in Sc-Microalloyed Al–Cu Alloy with Enhanced Creep Resistance. Mater. Today Nano.

[18] Chen B.A., Pan L., Wang R.H., Liu G., Cheng P.M., Xiao L., Sun J. (2011). Effect of Solution Treatment on Precipitation Behaviors and Age Hardening Response of Al–Cu Alloys with Sc Addition. Mater. Sci. Eng. A.

[19] Gao Y.H., Yang C., Zhang J.Y., Cao L.F., Liu G., Sun J., Ma E. (2019). Stabilizing Nanoprecipitates in Al-Cu Alloys for Creep Resistance at 300 °C. Mater. Res. Lett..

[20] Zhang H., Liu Y. (2023). Microstructures and Elevated Temperature Mechanical Properties of AlSi12Cu4Ni2 Fabricated by Laser Powder Bed Fusion. J. Manuf. Process..

[21] Sun T., Guo X., Xu R., Zhang Z., Zhang X. (2025). Enhancement of High-Temperature Properties of WA-DED 205 A Aluminum Alloy via the Addition of Nickel Element. J. Alloys Compd..

[22] Feng G., Dai H., Liu F., Zhao H., Jia H., Chang C., Ma C. (2025). Microstructural Evolution and High-Temperature Tensile Fracture Mechanisms of Al-Cu-Ni Alloy Weldments during Post-Weld Heat Treatment. Mater. Charact..

[23] Cheng B., Luo Z., Li G., Gong H., Liu X., Liu G., Yin F., Ma X., Zhao Y. (2025). High Fraction Al_3_BC-AlN Hybrid Reinforced Al Composites with High Modulus and Low CTE. J. Alloys Compd..

[24] Sun J., Huang Y., Liu W., Wu G., Qi F., Guo Y. (2024). Enhanced Ductility by Tailoring Precipitations in Micron TiB_2_ Reinforced Mg-Li Matrix Composites with High Modulus. Mater. Des..

[25] Zhou H., Ji Y., Wang Y., Feng K., Luan B., Zhang X., Chen L.-Q. (2024). First-Principles Lattice Dynamics and Thermodynamic Properties of α-, θ-, κ- and γ-Al_2_O_3_ and Solid State Temperature-Pressure Phase Diagram. Acta Mater..

[26] Hu K., Gao T., Liu G., Sun Q., Han M., Xu Q., Liu X. (2025). Comparative Evaluation of the Mechanical Properties of Al-Si-Cu-Ni-Mg Alloys with Distinct Spatial Architectures at Ambient and High Temperatures. Met. Mater. Int..

[27] Li G., Liao H., Zheng J., Chen H., Qian L., Yang M., Lu L., Shi M. (2022). Sc-Induced Great Increase in High Temperature Strength of Al-Si-Cu Heat-Resistant Alloy. J. Alloys Compd..

[28] Asghar Z., Requena G., Boller E. (2011). Three-Dimensional Rigid Multiphase Networks Providing High-Temperature Strength to Cast AlSi10Cu5Ni1-2 Piston Alloys. Acta Mater..

[29] He X., Lin B., Zhang W., Xiao H., Zhang W. (2022). Microstructures and Enhanced Mechanical Properties of (Al_3_Ti+Al_2_O_3_)/Al–Si Composites with Co-Continuous Network Structure Prepared by Pressure Infiltration. Ceram. Int..

[30] Li M., Gao T., Li C., Sun Y., Wu Y., Liu X. (2023). On the Nano–Treating Effect of Al_2_O_3_ on the Eutectic Si in Al–Si Alloy. Micron.

[31] Wang Y., Zhao X., Liu F., Hou X., Bai P., Cui X. (2025). Modification Mechanism of Eutectic Si Phases in Al-Si-Mg Series Alloys with Sc Addition. J. Alloys Compd..

[32] Li C., Li X., Zhang Y., Wen K., Xiao W., Li Y., Yu M., Gao G., Li Z., Xiong B. (2023). Effect of Sc Content on Microstructure Characteristics and Evolution of W Phase in Al–Cu–Li Alloys under as-Cast and Homogenization Conditions. J. Mater. Res. Technol..

[33] Yang X., Wang J., Li X., Xue C., Li Q., Miao Y. (2025). Achieving Ultra-High Performance after Thermal Exposure Temperature for Al-Cu Alloys by Stabilizing Nano θ′ Precipitates and Diversifying Micro-Compounds with Sc and Li. J. Alloys Compd..

[34] Qin J., Ma M., Tan P., Yi D., Wang B. (2022). Effects of Sc Alloying on the Evolution of Solidification Microstructure and Formation of W Phase in As-Cast 2519 Aluminum Alloys. J. Alloys Compd..

[35] Kairy S.K., Rouxel B., Dumbre J., Lamb J., Langan T.J., Dorin T., Birbilis N. (2019). Simultaneous Improvement in Corrosion Resistance and Hardness of a Model 2xxx Series Al-Cu Alloy with the Microstructural Variation Caused by Sc and Zr Additions. Corros. Sci..

[36] Dong H., Li J.F., Ma K.D., Xia F., Lei X., Liang Y.F., Guo Q.Q., Bai Y.P., Guo Y.C. (2025). Effects of Cooling Rate and Sc Content on Al_3_CuNi Phase in Cast Heat-Resistant Al–Si–Cu–Ni Alloys. J. Mater. Res. Technol..

[37] Dong H., Wang Y.Q., Guo Y.C., Ma C.H., Chen Q., Liang Y.F., Xia F., Ma K.D., Guo Q.Q., Zhang W.X. (2025). Optimizing the Phase Morphology and Creep Properties of Cast Heat-Resistant Al-Si-Cu-Ni Alloy via a Gradient Partial Remelting Treatment. J. Alloys Compd..

[38] Zhang N., Feng Y., Zhao S., Fu Y., Wang L., Guo E. (2024). Microstructure Evolution and Mechanical Properties of Al-12Si-xCu-yNi-1Mg Alloy with Different Ni/Cu Ratios. Mater. Today Commun..

[39] Li Y., Yang Y., Wu Y., Wang L., Liu X. (2010). Quantitative Comparison of Three Ni-Containing Phases to the Elevated-Temperature Properties of Al–Si Piston Alloys. Mater. Sci. Eng. A.

[40] Dimitrov D.M., Mincheva D., Slavov S.D. (2022). Influence of Porosity to Dynamic Young’s Modulus of Sintered Iron. Bayesian Approach. Mater. Today Proc..

[41] Käse H.R., Tesk J.A., Case E.D. (1985). Elastic Constants of Two Dental Porcelains. J. Mater. Sci..

[42] Suh J.-Y., Lee Y.-S., Shim J.-H., Park H.M. (2012). Prediction of Elastic Properties of Precipitation-Hardened Aluminum Cast Alloys. Comput. Mater. Sci..

[43] Nayak K.C., Kim S.-H., Lee J.-W., Bae D., Ahn J.-P., Lee K.-B., Choi H.-J. (2025). Advancing Giga-Strength and High-Modulus Aluminum Matrix Composites via Nitrogen-Induced Self-Forming Process. J. Mater. Res. Technol..

[44] Guo Y., Nie K., Deng K., Xu C. (2026). The Preparation of High Strength-Modulus-Thermal Conductivity GNPs-GFs/Mg-3Zn-0.1Y Composites by Liquid-Phase Dispersion and Low-Temperature Extrusion. Mater. Sci. Eng. A.

[45] Zhou J., Yun K., Qi L. (2025). Elastic Modulus in Magnesium Matrix Composites. RMR.

[46] Guo Y., Nie K., Deng K., Liu Z., Shi Q. (2024). Strength-Plasticity-Matched (GNPs+GFs)/Mg–3Zn-0.1Ymagnesium Matrix Composites with High Modulus through Liquid-Phase Dispersion and Multistep Deformation. Compos. Commun..

[47] Shin D., Shyam A., Lee S., Yamamoto Y., Haynes J.A. (2017). Solute Segregation at the Al/θ′-Al_2_Cu Interface in Al-Cu Alloys. Acta Mater..

[48] Jiang L., Rouxel B., Langan T., Dorin T. (2021). Coupled Segregation Mechanisms of Sc, Zr and Mn at θ′ Interfaces Enhances the Strength and Thermal Stability of Al-Cu Alloys. Acta Mater..

[49] Zhu R., Chen W., Chen Z., Sui Y., Qu Y. (2025). Effect of Combined Addition of Ni and Sc on Microstructure and High-Temperature Mechanical Properties of an Al-Cu-Mn Alloy. J. Alloys Compd..

